# Which endpoints should be applied in interventional trials? – From single uni-dimensional assessment tailored to a drug's mechanism of action to multi-component measures and multi-domain composites

**DOI:** 10.1016/j.ostima.2024.100256

**Published:** 2024-12-25

**Authors:** Felix Eckstein, Tanja Stamm, Jamie Collins

**Affiliations:** a Research Group for Musculoskeletal Imaging, Center for Anatomy and Cell Biology, Paracelsus Medical University, Salzburg, Austria; b Ludwig Boltzmann Institute for Arthritis and Rehabilitation, Vienna, Austria; c Chondrometrics GmbH, Freilassing, Germany; d Section for Outcomes Research, Center for Medical Statistics, Informatics, and Intelligent Systems, Medical University of Vienna, Vienna, Austria; e Brigham & Women's Hospital, Harvard Medical School, Boston University, MA, USA

**Keywords:** Endpoint, Osteoarthritis, Image assessment, Composite measure, Multi component measure, Mechanism of action (MOA)

## Abstract

**Objective:**

A vast array of structural/imaging and clinical endpoints/outcomes are available today to osteoarthritis epidemiologists or trialists. Which assessments are best suited for which studies remains unsettled. When several assessments are available, these may be analyzed together (simultaneously or hierarchically), using statistical modeling and adjustment. Or, alternatively, they may be combined to form more complex multi-component or composite (potentially multi-domain) endpoints/outcomes. This review describes such concepts and their challenges, using examples from current osteoarthritis imaging research.

**Design:**

A narrative, non-systematic literature search (PubMed and others) was conducted, and informal consultations were held with experts in the field. The identified concepts and experimental findings were then organized to present an integrated framework.

**Results:**

Single imaging assessments can encompass one (uni-dimensional) or more (multi-dimensional) structures. Integration of image assessments of one structure/tissue across anatomical locations provides aggregate measures. This can also be created across heterogeneous (multi-dimensional) types of assessments (multi-component/composite), either within an area (such as imaging - single domain) or across broader areas of health and well-being (multi-domain). Weighting, standardization, and (clinical) usefulness are crucial characteristics of multi-component/composite endpoints. Examples of these concepts are here provided in the context of osteoarthritis imaging.

**Conclusions:**

Options for multi-component/composite endpoints in osteoarthritis research are virtually infinite. Smart research strategies are required to explore and validate these vast possibilities, with appropriate statistical treatment being paramount. A one-size/endpoint-fits-all approach will likely fail in observational and interventional studies. Imaging assessment needs to be tailored to both the drug's unique mechanism of action, and to the participants’ morpho-type.

## 1 Introduction

With the advent of more advanced technology for imaging acquisition and analysis in osteoarthritis (OA) research several decades ago [1,2], the number and types of structural assessments available to OA experimentalists, epidemiologists and trialists has risen dramatically. Today, the number by far exceeds that of the well-established clinical measures (of pain, function, and joint-related quality of life). These days researchers can thus select from an overwhelming, but sometimes also confusing, wealth of imaging assessments to characterize joint morpho-types [3], and the impact of interventions on structural pathology. Yet, it is a matter of ongoing debate which (or which combination) of image assessments should preferably be used in various experimental, observational or interventional studies. If more than one type of assessment is desired as study endpoint, these may be looked at together, simultaneously or hierarchically, yet remaining separate entities [4]. In this case, an a-priori defined hierarchical statistical analysis plan, specifying the order of statistical testing (primary and secondary endpoints) as well as adjustment for multiple comparisons, is necessary [4]. Alternatively, multi-component or composite endpoints may be used [5], where these assessments are merged to form novel measures, potentially spanning across broader areas of health (domains).

The current review bases on an invited presentation delivered at the 18th International Workshop of OsteoArthritis Imaging (IWOAI) in June 2024. It aims to provide a summary and framework on single vs. multi-component /composite imaging assessment. We introduce terminology and present theoretical concepts and challenges of this research area, giving examples from OA imaging science.

## 2 Methods

A non-systematic literature search (PubMed) was conducted, starting with a list of terms from the title of the presentation at the IWOAI, and search terms from the OA imaging field. These terms included (non-exclusive list): “imaging”, “quantitative”, “joint”, “whole organ”, “joint tissue”, “joint structure” (cartilage, meniscus, bone, bone marrow, synovium, etc.), “assessment”, “score”, “measure”, “endpoint”, “outcome”, “intervention”, “trial”, “DMOAD”, “uni-dimensional”, “multi-dimensional”, “multi-component”, “composite”, “domain”, “multi-domain”, “drug”, “mechanism of action”, “phenotype”, “morpho-type” and others. Additional conceptional information was obtained using Google and Chat GPT 4.o. Finally, to receive broad and detailed input, informal (non-structured) consultations were held with experts in various research areas, before and during the 18th IWOAI (June 2024; Marrakech). The identified terminology, experimental findings, concepts, and recommendations were then organized into a systematic framework, suggesting a classification of terms, and providing examples from current OA imaging science.

## 3 Results

### 3.1 Definition of terms

Evaluation of images (“assessment”) in medicine is commonly performed using “scores” or “measures” (Table 1). A “score” thereby represents a discrete numerical value assigned to describe an observation within the context of a standardized system, typically based on evaluation by an expert or algorithm. Although “grades” represent categorical classifications describing the severity of imaging findings (staging) and represent a parameter of “classification” rather than “quantification” (score), both terms are mostly used interchangeably in imaging. They are represented by ranked, categorial, ordinal values. Examples are the Kellgren Lawrence (KL) [6] or joint space narrowing (JSN) grade [7] in the radiographic evaluation of knee OA, where grade 2, for instance, does not at all represent twice the damage observed in grade 1. Another example is the whole-organ MRI Osteoarthritis Knee Score (MOAKS), used in the semi-quantitative evaluation of knee joint structural pathology [1]. A measure, in contrast, represents the product of a fully quantitative process of data collection (measurement), representing a continuous value. Examples covering different dimensionalities are cartilage thickness (mm) [8], total and denuded subchondral bone area (cm^2^) [8], or cartilage or bone volume (cm^3^) [2]. Measures may actually be converted into grades, using thresholds or cut-points. The body mass index, for instance (a continuous value) often is converted into ordinal grades of underweight, normal weight, overweight, and obese using thresholds of 18.5, 25, and 30, respectively. In OA trials, a continuous measure of longitudinal change in JSW may be converted to progressors (>0.7 mm) or non-progressors (≤0.7 mm) [9]. A metric represents a specific variable, related to the performance or quality of a score, grade, or measure (Table 1). Examples are validity/accuracy, reliability/precision [10] (repeatability and reproducibility); the Dice similarity coefficient (segmentation agreement), standardized response means (sensitivity to change), responsiveness to treatment (effect size, for instance by Cohens’ D), and others. For further reviews of definitions and the evaluation of quantitative performance measures by the Quantitative Imaging Biomarker Association (QIBA) see [11,12]. Here, we will use the term “score/grade” when describing discrete and semi-quantitative variables, and “measure” when referring to continuous, fully quantitative measures. The term “assessment” will be used “neutrally” when addressing either semi-quantitative scores or quantitative measures.

**Table 1:** Terms frequently used in context with the assessment of medical images in clinical studies.

Term	Short explanation (for longer explanations see full text)
Assessment	Evaluation of images by either scores or measures
Score / Grade	Discrete numerical value assigned to an observation (ranked, categorial, ordinal values)
Measure	Product of a quantitative process of data collection (continuous value)
Metric	Variable related to the performance or quality of an assessment
Biomarker	Characteristic indicative of a normal biological or pathological process, or a response to an exposure
Endpoint	Biomarkers used to evaluate scientific relationships or the efficacy of an intervention
Outcome	Biomarker representing a broader aspect of health (feel, function, survive)
Surrogate outcome	Biomarker that has been qualified to substitute a direct measure or an outcome

Image assessments can adopt the role of a “biomarker”, a defined characteristic indicative of a normal biological process, of a pathological process, or of a biological response to an exposure, for instance a therapeutic intervention [13]. When biomarkers are used to evaluate scientific relationships (i.e. risk factors) in an epidemiological study, or to interrogate the efficacy of an intervention in a clinical trial, they may be termed “endpoint” (Table 1). Change in cartilage volume, thickness, bone size, bone shape, and others, have, for instance been used as endpoints in clinical trials of DMOADs [2]. When such a measure represents a broader aspect of a participant's health, specifically how a patient feels, functions, or survives (or her/his joint survives) it fulfills the requirements of being an “outcome”. Hence imaging assessments can only represent endpoints, but never outcomes. However, if appropriately qualified (validated), an imaging biomarker can assume the role of a “surrogate outcome” and may act as substitute of a direct assessment of a clinically meaningful outcome [14] (Table 1). Surrogates are extremely versatile and useful because they are generally more straight-forward, and often more objectively measured; often also they respond earlier to disease progression or to a therapeutic intervention than clinical outcomes. However, even if surrogate outcomes “correlate” with clinical outcomes, they do not necessarily predict these reliably, and therefore must be qualified. Generally, this must occur “de novo” in every specific context and novel application, pertinent to different modes of action (MOA) of new drugs [15]. Although OA imaging assessments commonly serve as endpoints in clinical trials, the FDA has not accepted any of them as a “surrogate endpoints” for evaluating efficacy of disease modifying osteoarthritis drugs (DMOADs) in their 2018 Guidance document (https://www.fda.gov/regulatory-information/search-fda-guidance-documents/osteoarthritis-structural-endpoints-development-drugs). This applies neither generally nor in any particular context, and thus represents a great current obstacle to an efficient DMOAD development process.

### 3.2 From uni-dimensional to multi-component/composite multi-domain endpoints

Single, uni-dimensional (or uni-variable) assessment represents one structure/tissue or entity only (Table 2). Examples are an anterior cruciate ligament (ACL) “score” (normal vs. complete tear) [16], or a “measure” (continuous scale) of meniscus extrusion distance in a single slice, or extrusion area across several MR images (Table 2) [17]. Yet, they can also be more complex, from either a segmentation point of view (e.g., bone marrow lesion (BML) volume [18,19] or in the way they capture the intricate shape of an anatomical structure such as the bony metaphysis in a single continuous measure, e.g., the B-score [2,20]. Examples of clinical single, uni-dimensional assessments in OA are the numerical rating scale for pain (NRS; a score), or the chair stand test for physical function (CST; a measure), both used, for instance, in the Osteoarthritis Initiative [21] (Table 2).

**Table 2:** Classification of different types of assessments (scores or measurements) in osteoarthritis (OA) research: Short explanation of category, and examples given from various domains, including imaging (structure), pain, function, and surgery.

Type of assessment	Short explanation	Examples
Single uni-variable	Single assessment capturing 1 entity (single location)	Anterior Cruciate Ligament (ACL) tear#
		Meniscus extrusion distance or area
		Bone Marrow Lesion (BML) volume
		B-Score (Bone shape)
		Numerical rating scale (NRS) pain
		Chair stand test (functional performance)
Single multi-variable	Single assessment capturing > 1 entity (single location)	Joint space narrowing (JSN) score
		Joint space width (JSW) measurement
		Effusion synovitis (ES) volume
Aggregate (uni-variable)	Assessment capturing the same type of entity at multiple locations (same joint); various ways of integrating data	Osteophyte score
		MRI Osteoarthritis Knee Score (MOAKS): *Cartilage damage, or meniscus morphology/position,or bone marrow lesions/cysts, or Hoffa / Effusion synovitis*
		Cartilage thickness
		Cartilage composition: *T2 or T1rho or dGEMRIC or GAGCest, or Sodium, etc.*
		Synovial thickness
Multi-component: multi-dimensional single-domain	Assessment integrating several entities belonging to the same broader area of health/well-being (domain)	Kellgren Lawrence Grade (KLG)
		MOAKS total Score [or WORMS or BLOKS total score]
		Western Ontario & McMaster (WOMAC) pain or function
Multi-component: multi-dimensional multi-domain	Assessment integrating several entities belonging to different broader areas of health/well-being (domain)	American College of Rheumatology (ACR) definition of OA
		Western Ontario & McMaster (WOMAC) total score
		Knee Injury and Osteoarthritis Outcome Score (KOOS)
		Virtual knee replacement (KR)*
		FNIH OA Biomarker Consortium Progressor Definition
Composite: single-domain	Assessment marked positive if any single of several entities from the same domain is positive	“Any surgery”, (e.g., meniscectomy or KR)
Composite: multi-domain	Assessment marked positive if any single of several entities from different domain is positive	Joint “death” defined as virtual KR or KR OA progression, either structure or pain

# associated bone marrow lesion/cyst at the site of insertion/origin or repair status may also be evaluated; *some definitions only include pain variables: then this is a multi-component; multi-dimensional, single-domain assessment

Minimum (medial) radiographic joint space width (JSW) is considered a simple, one-dimensional measure for assessing OA radiographic disease status and structural progression. Yet, it is multi-dimensional (multi-variable) in nature, as it is governed by at least two structures, i.e. by femoro-tibial cartilage thickness, and by meniscus pathology/extrusion. Both make relevant contributions to JSW cross-sectionally [22] and longitudinally [23] with joint positioning vs. the film and weight-bearing being further influential methodological factors [24]. Similar considerations apply to the radiographic JSN grade for the reasons mentioned above, and to synovitis effusion (SE), because in inter-mediate or proton density weighted spin-echo sequences the inflamed synovium and the (ample) synovial fluid both appear hyper-intense and are therefore visually indistinguishable [25] (Table 2). Single, multi-dimensional assessments are sometimes addressed as “composite” measures, because more than one factor governs the target assessment; yet, this is inviting confusion, because there are stricter definitions of “composites”, which we will address below.

If several assessments are taken of the same structure, e.g., across different regions (or of the same clinical entity), this may be considered an aggregate. An aggregate assessment is always uni-dimensional (Table 2). Different scores or measures are typically combined using sums, maxima, averages/means, medians, standard deviations, etc. Examples of aggregate scores are those used for osteophyte scores around the knee by MRI [26], or those for cartilage lesions [27], meniscus lesions [28], or synovitis (effusion or Hoffa) [29] (Table 2). Examples of aggregate measures are “cartilage thickness” determined by MRI-based cartilage morphometry, typically encompassing 1000 data points per square centimeter of the joint surface [2,30]. Other examples are cartilage transverse relaxation time, T2 [31,32] (or other compositional quantitative (q)MRI measures in the deep and superficial cartilage zone), and “synovial thickness” by contrast-enhanced (CE) MRI [25,33]. Within-measure summaries can be complex, for instance when using longitudinal regional variability to guide the integration process [34]. This strategy is useful to determine the overall magnitudes of cartilage loss and cartilage gain, independent of their regional distribution and associated biomechanical determinants/constraints [35]. Local variation in MRI relaxometry signals can be employed to create complex summaries, which potentially relate better to tissue properties and relevant clinical conditions than means or standard deviations. These include texture analysis [[36], [37], [38], [39]], statistical parametric mapping [40,41], the spatial gradient method [42,43], principal component analysis of Z-score difference maps [44,45], and others.

The terms "multi-component" and "composite" assessment are applicable when the evaluation is multi-dimensional (covering >1 structure/tissue, or clinical entity). In contrast to (the above) single, multi-dimensional measurements, such as JSW or ES, the components are not intricate to another by laws of nature, but represent separate scores or measures that contribute to a common endpoint along the rules defined by a scientist (Fig. 2). These components can remain within a single domain (such as pain, or functional disability, or imaging, or surgical intervention, etc.), or may extend across these broader areas of health and well-being (multi-domain). The combination of imaging endpoints obtained from different modalities (such as MRI and CT, or MRI and PET) sometimes is addressed as a “multi-domain” approach; however, we consider this combination “multi-modal”, as it spans across various imaging technologies, but nevertheless remains within the broader scope of medical imaging. A “composite” endpoint is defined in a way that only one (of the several) components needs to reach a specified threshold so that the entire endpoint becomes "positive", irrespective of the status of the other components [46,47] (Fig. 2). An analogy from professional sports is the “Tricot jaune” at the Tour de France; this is granted to the fastest rider in the overall standings, independent of how his team mates are ranked. A medical example from the cardiovascular field is the “major adverse cardiac event” (MACE) as composite outcome, typically including either cardiovascular death, or non-fatal acute myocardial infarction, or non-fatal stroke, or revascularization [48]. Yet, if the components are combined to a novel score, and a clear definition is provided above which threshold this score will be “positive”, this may also be considered a composite [46,47]. “Multi component” endpoints, in contrast, become “positive” when certain combinations of components meet some pre-determined thresholds, in certain pre-set combinations [5] (Fig. 2). Whilst each component contributes a separate piece of information, the components remain separate and distinguishable throughout (Fig. 2). Analogies from sports are the team time trials at the Tour de France, where time counts when the 4th (of 8) cyclists reaches the finish line. The standards by which components in multi-component endpoints (or composites) are aggregated, weighted and standardized are not defined. Therefore, these principles must be established, explored, and validated empirically, best by testing clinical usefulness. The QIBA outlined that when quantitative imaging biomarkers (QIBs) are combined to multi-parametric QIBs (multi-dimensional, multi-component endpoints) obtained from one or several imaging modalities, additional clinical utility may be drawn over single measures for detecting disease, identifying phenotypes, detecting longitudinal change, predicting outcomes, or other potential intended uses [49]. The QIBA proposed that, if carefully selected, each QIB can contribute unique clinical information, with the value of the multi-parametric QIBs exceeding those of their individual components [49].

**Fig. 1:**
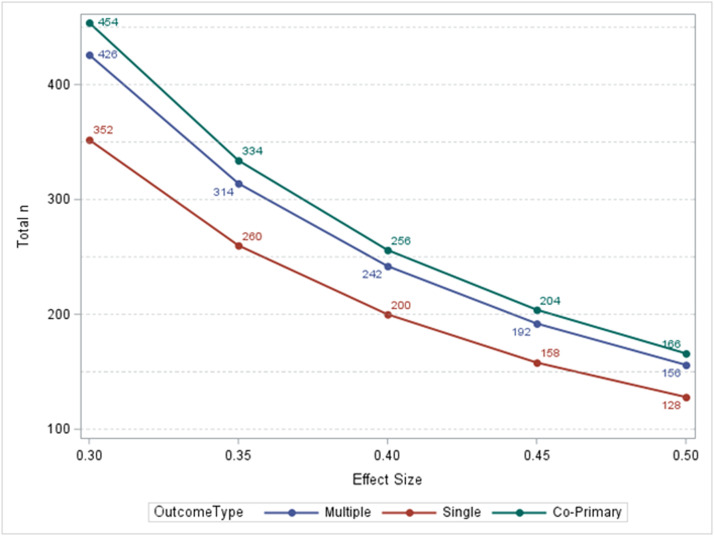
Sample size required for a given effect size for a single endpoint (red), multiple endpoints (blue) and a co-primary endpoint (green).

**Fig. 2:**
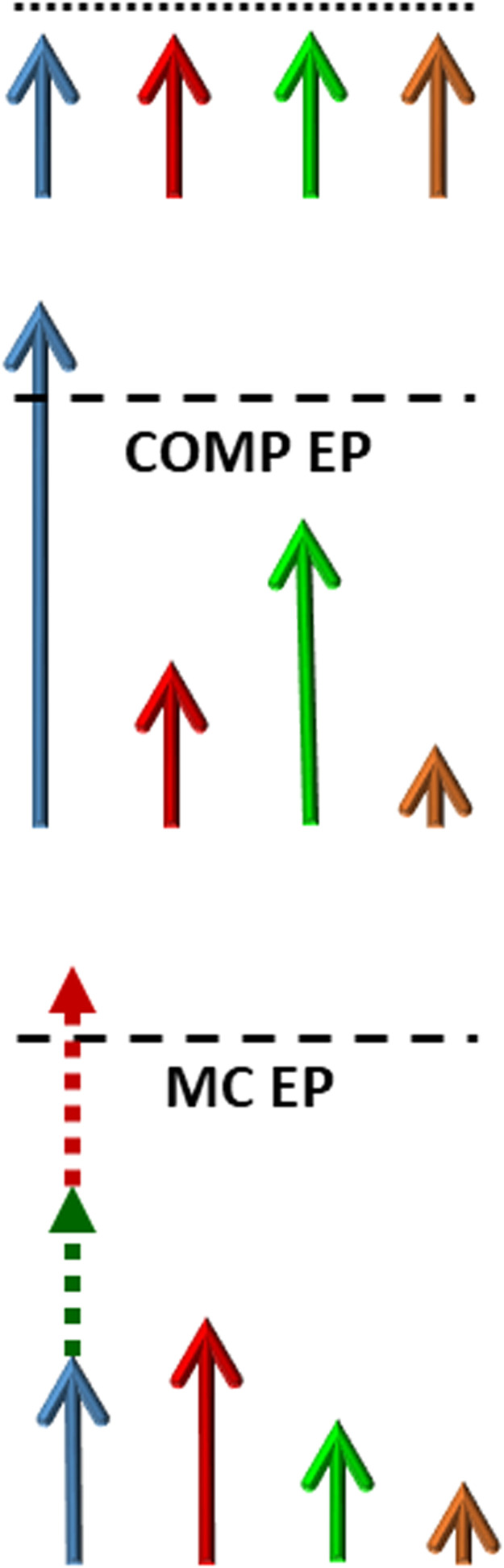
Schematic visualization of multiplicity approaches of endpoints (EP): A) Multiple independent endpoints that require statistical adjustment when tested in parallel to become positive. B) COMP EP = composite endpoint. Only one of several defined endpoints (here blue) has to pass a previously defined threshold, for the COMP EP to become positive (no statistical adjustment required). C) MC EP = multiple component endpoint. All endpoints contribute based on previously defined mathematical relationships. If passing the threshold according to this algoithm, the MC EP becomes positive (no statistical adjustment required).

A good example of a multi-component, multi-dimensional, single domain endpoint is the Kellgren Lawrence grade (KLG), reflecting radiographic bone pathology (osteophytes, subchondral bone sclerosis and attrition) and JSN (cartilage and meniscus pathology) [22,23]. Another example from the radiology field is the whole-organ MRI-based MOAKS reading system that permits evaluating structural pathology in different joint regions and different imaging/pathology features for most joint tissues (multi-dimensionality) [1,16,50]. This provides a plethora of potential different combinations, with the opportunity to characterize various morpho- /pheno-types [3,51] of the disease that may require different medical treatments, but also with challenges in how to integrate in the vast amount of readings available [51]. Multi component endpoints derived from quantitative imaging measures have been probed for identifying types that relate to incident [52] or progressive [53] knee OA. Based on 12 potential composite qMRI candidates, Harkey et al. [52] defined a “disease activity” multi-component endpoint (sum of BML and effusion synovitis volume) and a “cumulative cartilage damage metric” (obtained from several local cartilage thickness measures) and found that both were associated with accelerated incident radiographic knee OA over 2 years. In a later study [53], the authors tested the construct validity of qMRI composite candidates in predicting disease progression. Whereas the “disease activity metric” was somewhat more strongly related to symptomatic progression (WOMAC pain), the” cumulative cartilage damage metric” tended to show a stronger relationship with radiographic (KLG and medial JSW) progression [53]. WOMAC pain (or function) scores also represent multi-dimensional, single domain multi component endpoints, each collecting clinical information on the same domain, but for variable conditions, such as pain or functional limitations during different tasks and activities (Table 2). Again, if assessment integrates across broader areas of health and wellbeing, such as imaging (structure) and clinical information (pain or function) these are considered “multi-domain”. Examples for such a construct are the American College of Rheumatology (ACR) definition of OA, which is based on clinical symptoms, physical examination, and radiographic findings [54]. As typical for multi-component endpoints/outcomes, each of these “domains” provides a separate piece of information, contributing to the overall diagnosis [54]. The Foundation for the National Institute of Health (FNIH) Osteoarthritis Initiative (OAI) Biomarker Consortium definition of “disease progression, for instance, is based on meeting a certain (>0.7 mm) change in radiographic JSW as well as a change in WOMAC pain over year 2–5 follow-up” [9,55,56], a typical characteristic of a multi-domain multiple-component outcome. Had a different definition been chosen, such as labeling participants with pain progression or radiographic progression (or both) as progressors, this would equate to a multi-domain composite outcome [9,55,56]. Another example is virtual knee replacement (vKR), where a definition of “endstage” knee OA that requires replacements was based on pain (function) and radiographic status [57,58].

Examples of composite assessments that remain within a single domain are “any surgery” endpoints, where, for instance, either meniscectomy or knee replacement count as events rendering the outcome positive [59] (Table 2). Other examples in OA research are a definition of “joint death” indicated by either “real KR” or “virtual KR” [60], or “real KR or severe symptom state” [14].

### 3.3 Statistical considerations

When more than one (single or aggregate) assessment is to be included in the overall evaluation, a predefined statistical analysis plan needs to define the endpoint hierarchy (primary, [co-primary], key secondary, secondary, [co-secondary], exploratory, etc.), each with certain statistical considerations for multiple comparisons [4]. Co-primary endpoints require that treatment effects be demonstrated for all selected endpoints, and therefore require adjustment of statistical power for each individual test to ensure the overall power is maintained (Fig. 1). Using a multiple endpoint approach, a treatment effect needs to be demonstrated on at least one endpoint, requiring an adjustment of alpha for each test to ensure overall Type I error is maintained (Fig. 1). For example, to detect a 0.5 effect size with an overall type I error of 0.05 and an overall power of 80 %, n = 64 participants are required per group using a single primary endpoint, n = 78 using two multiple endpoints, and n = 83 using two co-primary endpoints.

Because multiple-component or composite endpoints combine assessments into one (single) endpoint, there is no statistical penalty for multiplicity, as the “inherent” components are not tested in parallel. However, it has to be borne in mind that the “factors” that serve as components remain in a “black box”. If an intervention affects various components of the endpoint differently, there is a risk of masking of the treatment effect [61,62]. While it is possible to disentangle the effects of treatment on individual components of the outcome, these analyses must be considered as exploratory and/or undergo proper adjustments for multiple testing, and require a thoughtful analytic approach [62]. Composite endpoints may be defined as the incidence of any individual component, i.e., KR or vKR [60], which considers each endpoint as being of equal clinical importance; however, it must first be established whether this is appropriate [63]. Yet, algorithms that weigh individual components also require thorough validation [5].

### 3.4 Application of these concepts to interventional trials

Attempts have been made to define, qualify and validate a single imaging endpoint or outcome measure as being optimal for all types of OA trials, and in convincing regulatory agencies to approve such an endpoint universally. Yet, this is hardly realistic or reasonable, given differences in the MOA between drugs, and differences in patient cohorts with matching morpho- or pheno-types.

In a randomized clinical trial (RCT) on a putative anabolic cartilage agent (FORWARD), an aggregate measure of mean cartilage thickness change across the (medial and lateral) weight-bearing femorotibial joint - the primary (structural) endpoint - showed a statistically significant difference between participants treated i.a. with 100 µg Sprifermin vs. those injected with placebo, whereas the single, multivariable measure of radiographic JSW did not [64]. This is most likely due to a) greater robustness and sensitivity of an aggregate measure that integrates information across the entire joint compared with a single point measure, b) greater specificity of an MRI based measure of cartilage to the MOA of the anabolic cartilage drug than a multi-dimensional measure that depends both on cartilage and meniscus status, and c) the fact that a single (uni-dimensional) measurement is more strongly dependent on the image acquisition conditions (orientation of the film relative to the joint) than one that is three-dimensional. Yet, a measure of cartilage thickness (or volume) is unable to differentiate to what extent disease-related cartilage thinning in the joint has been affected, or just cartilage thickening has been boosted, potentially at anatomical locations where such an increase in cartilage thickness is biomechanically and functionally not useful. Yet, separating the measure of thickness change from its specific anatomical location, and creating aggregate measures of thickening and thinning revealed that not only the cartilage thickening was enhanced by the drug, but that cartilage thinning was substantially reduced, wherever it may have occurred in any joint. Surprisingly, this reduction was almost the level of that observed (methodologically) in healthy reference subjects [35]. Further, in a DMOAD trial the type of assessment also needs to match the patient morpho-/pheno-types [3], and hence the respective patient selection criteria. Post-hoc analyses of the FORWARD study revealed that the effect of Sprifermin on cartilage was similar for patients with thinner baseline cartilage (less JSW) and greater baseline pain than for those with greater cartilage thickness and less pain [65]. This was opposite to the often-made assumption that a therapeutic effect can only be expected at earlier stages the disease. And all the more, in participants with less cartilage thickness and more pain the treatment-related positive effect on structure did in fact translate into a meaningful clinical benefit (vs. placebo), whereas in those with thicker baseline cartilage and less baseline pain in the FORWARD study, a treatment-response to pain was not seen [65]. From a patho-physiology standpoint it makes sense that participants who display signs of net cartilage matrix loss at baseline will profit more symptom-wise from anabolic cartilage treatment, because an existing loss of cartilage at baseline suggests that the tissues may be structurally involved in the disease, and an anabolic reversal of that status may improve its condition [65]. Other DMOAD trials also have attempted to match the MOA and the patient inclucion criteria with the measurement endpoints, including those targeting modification of cartilage [[66], [67], [68], [69]], synovitis [[69], [70], [71]], BMLs [[72], [73], [74]], or bone (shape) [75,76]. In cases of synovitis and BMLs, it is an open question on whether demonstrating a positive effect on structure will be sufficient to receive DMOAD approval from a regulatory agency; synovium and bone marrow represent strongly reactive joint tissues and fluctuations of their status are seen in normal disease progression [[77], [78], [79]]. Structurally, they are not directly involved in load-bearing and biomechanical functioning, and most of the above trials (therefore) have examined cartilage at least as a secondary endpoint. Certain degradation products of the cartilage are known to cause synovitis and to have negative downstream effects on other joint tissues that are deleterious to joint health and enhance progression [80]. Hence, DMOAD trials may use the opportunity to assess synovitis or BMLs as primary, and cartilage (or other weight-bearing tissues) as co-primary or secondary structural endpoints. Alternatively, both (or more) structural dimensions may be included in multi-component, multi-dimensional, single domain (imaging) endpoint, or could be combined further with measures of pain and/or function to form a multi-component, multi-domain outcome. Yet, more is not necessarily better, and important considerations are to what extent the components combined a) are correlated (providing redundant information), b) change in the same or different directions and potentially cancel each other out, c) fluctuate in sync or independently, and d) are precise (low test-retest error). As mentioned previously, multi-component endpoints and composites need to be thoroughly validated clinically, best repeatedly and de novo in each new drug-development context.

Applied to late stage OA, multi-component endpoints may be created as a composite of knee replacement (KR) or severe clinical (symptom) state, the latter defined by conservative thresholds of patient-reported outcomes (PROs) [14]. Assessments of severe clinical state conditions may be combined to a multi-component outcome of virtual KR (vKR), either including or not including radiographiy [57,58,81]. In terms of validation, they should display clinical utility, i.e. be related to KR [57,58], or should be similarly related to relevant predictors of KR [60]. The attractiveness of such an approach is that regulatory agencies are very focused on outcomes such as KR (or vKR), as these are very closely related to how a patients feels, function or her/his knee survives (i.e. receives surgery) [14]. A multi-domain composite defined as “KR or vKR” may be powerful, as the vKR component may partially compensate for the elective nature of KR. The number of KRs is limited by the availability of surgical intervention in a given healthcare system, the socio-economic status of the patient or country, the educational status of the patient, the unwillingness or un-ability of the patient to undergo surgery, and further constraints. A recent estimate showed that using a composite KR or critical thresholds of pain and function outcomes, rather than KR alone, reduces required sample sizes by 40 % [14], albeit the estimated sample sizes for OA confirmatory trials of greater than 2000 participants are still quixotic and barely feasible for clinical development by industry sponsors.

If a study is performed at earlier disease stages, for instance when testing physiotherapy or DMOADS, that aim to prevent disease progression in patients with meniscus injury, an “any surgery” composite outcome [59] can be used effectively to explore whether the intervention prevents surgery. If the study is targeted at more long-term effects of the therapy, single or multi-component imaging endpoints can be used, but these would have to be previously qualified as surrogate biomarkers that eventually predict KR (or a KR/vKR composite [60]).

## 4 Conclusion and outlook

Multi-component endpoints are seductive given the level of data integration achievable across different dimensions, modalities, and domains. However, the components remain in a “black box” and attempts to unravel their individual contribution statistically comes with threats to type I error and/or power. Multi-component endpoints may thus be more suitable for interventional studies seeking regulatory approval than for research applications that try to unravel risk factors, proof of (therapeutic) concepts, or other exploratory relationships in OA research. The plethora of available endpoints in OA research is daring, and the number of options almost infinite. Therefore, smart research strategies need to explore and validate these gigantic possibilities, using an appropriate statistical framework. A one-endpoint-fits-all approach must fail, because (imaging) assessment needs to be tailored to the participants’ morpho- and/or pheno-type, and to the putative DMOAD's mechanism of action (MOA). Integrative assessments across dimensions (and domains) have great potential, but in each case this needs to be balanced against the loss of information how each component contributes to the overall response.

## Consent for publication

All authors have consented publication

## Authors' contributions

• Study conception and design: FE
• Acquisition of data: FE, JC
• Analysis & interpretation of data: All authors
• Writing of the first manuscript draft: FE
• Critical manuscript revision and approval of final manuscript: All authors

## Role of the funding source

The authors did not receive funding for writing this article or activities associated with its making.

## Ethics approval

No original data is published in this article

## Declaration of competing interests

The authors declare the following financial interests/personal relationships which may be considered as potential competing interests: CONFLICTS: • Felix Eckstein is CEO/CMO and co-owner of Chondrometrics GmbH; he has provided consulting services to Merck KGaA, Kolon-Tissuegene (KTG), Servier, Galapagos, Novartis, 4P Pharma, Formation Bio, and Peptinov outside the submitted work. • Jamie Collins provides consulting services to Boston Imaging Core Labs, LLC and is on the Scientific Advisory Board of Chondrometrics GmbH. • Tanja Stamm has received grants and consulting fees from AbbVie, Roche, Sanofi, Takeda, and Novartis outside the submitted work.
